# Genome Characterization and Phylogenetic Analysis of a Novel Endornavirus That Infects Fungal Pathogen *Sclerotinia sclerotiorum*

**DOI:** 10.3390/v14030456

**Published:** 2022-02-23

**Authors:** Xin Luo, Daohong Jiang, Jiatao Xie, Jichun Jia, Jie Duan, Jiasen Cheng, Yanping Fu, Tao Chen, Xiao Yu, Bo Li, Yang Lin

**Affiliations:** 1 State Key Laboratory of Agricultural Microbiology, Huazhong Agricultural University, Wuhan 430070, China; xinluo@webmail.hzau.edu.cn (X.L.); daohongjiang@mail.hzau.edu.cn (D.J.); jiataoxie@mail.hzau.edu.cn (J.X.); jiajichun@webmail.hzau.edu.cn (J.J.); taochen@mail.hzau.edu.cn (T.C.); 2 The Provincial Key Lab of Plant Pathology of Hubei Province, College of Plant Science and Technology, Huazhong Agricultural University, Wuhan 430070, China; duanjie@webmail.hzau.edu.cn (J.D.); jiasencheng@mail.hzau.edu.cn (J.C.); yanpingfu@mail.hzau.edu.cn (Y.F.); xiaoyu@mail.hzau.edu.cn (X.Y.); boli@mail.hzau.edu.cn (B.L.)

**Keywords:** endornavirus, *Sclerotinia sclerotiorum*, SsEV11, gammaendornavirus

## Abstract

Endornaviruses are capsidless linear (+) ssRNA viruses in the family *Endornaviridae*. In this study, Scelrotinia sclerotiorum endornavirus 11 (SsEV11), a novel endornavirus infecting hypovirulent *Sclerotinia sclerotiorum* strain XY79, was identified and cloned using virome sequencing analysis and rapid amplification of cDNA ends (RACE) techniques. The full-length genome of SsEV11 is 11906 nt in length with a large ORF, which encodes a large polyprotein of 3928 amino acid residues, containing a viral methyltransferase domain, a cysteine-rich region, a putative DEADc, a viral helicase domain, and an RNA-dependent RNA polymerase (RdRp) 2 domain. The 5’ and 3’ untranslated regions (UTR) are 31 nt and 90 nt, respectively. According to the BLAST result of the nucleotide sequence, SsEV11 shows the highest identity (45%) with Sclerotinia minor endornavirus 1 (SmEV1). Phylogenetic analysis based on amino acid sequence of RdRp demonstrated that SsEV11 clusters to endornavirus and has a close relationship with *Betaendornavirus*. Phylogenetic analysis based on the sequence of endornaviral RdRp domain indicated that there were three large clusters in the phylogenetic tree. Combining the results of alignment analysis, Cluster I at least has five subclusters including typical members of *Alphaendornavirus* and many unclassified endornaviruses that isolated from fungi, oomycetes, algae, and insects; Cluster II also has five subclusters including typical members of *Betaendornavirus*, SsEV11, and other unclassified viruses that infected fungi; Cluster III includes many endorna-like viruses that infect nematodes, mites, and insects. Viruses in Cluster I and Cluster II are close to each other and relatively distant to those in Cluster III. Our study characterized a novel betaendornavirus, SsEV11, infected fungal pathogen *S. sclerotiorum*, and suggested that notable phylogenetic diverse exists in endornaviruses. In addition, at least, one novel genus, Gammaendornavirus, should be established to accommodate those endorna-like viruses in Cluster III.

## 1. Introduction

Endornaviruses are a group of virionless viruses with linear, (+) ssRNA genome. The genome size of endornaviruses is various, with a range of 9.7–17.6 kb [1,2]. With very rare exceptions [3], endornaviruses typically have a large open reading flame (ORF) that encodes a polyprotein with methyltransferase (MTR), helicase (Hel), glycosyltransferase (GTR), and RNA-dependent RNA polymerase (RdRp) [2]. Endornaviruses are phylogenetically related to alpha viruses, and have been grouped in the Family *Endornaviridae*; currently, two genera, namely *Alphaendornavirus* and *Betaendornavirus*, have been established in this family based on their genome size, host, and the presence of unique domains [4,5,6]. Viruses that belong to *Alphaendornavirus* were identified from plants, fungi, and oomycetes [7,8,9,10,11,12,13], while viruses in genus *Betaendornavirus* infect fungi only [2]. Recently, endorna-like viruses were also found in animals, such as nematodes, mites, and insects [14,15,16,17], but their taxonomic status has not been determined.

*Sclerotinia sclerotiorum* belongs to ascomycetes, it is a cosmopolitan plant pathogen that can attack more than 700 plant species, mainly dicotyledons [18]. It can destroy lots of economic important crops that belong to crucifer, legume, compositae, Solanaceae and so on, while it could also endophytically grow on wheat, rice, and other Poaceae plants [19]. Hypovirulent viruses infecting *S. sclerotiorum* was potential biocontrol factors. For instance, the infection of two viruses separately, SsHV1 and SsDAHV-1, reduces the pathogenicity of *S. sclerotiorum* apparently [20,21,22]. It is very important to mine more valuable biocontrol factors [23]. In addition, about 16 families or genera of +ssRNA, -ssRNA, dsRNA, and ssDNA viruses are reported in *S. sclerotiorum* [24,25,26,27]. The diversity of viruses is very rich. *S. sclerotiorum* is an ideal material for studying viral evolution and abundance. Endornaviruses also have been isolated from *S. sclerotiorum*, among them, Sclerotinia sclerotiorum endornavirus 1 (SsEV1) is a worldwide virus, which has been found in Asia, North American, and Oceania [6,24,28,29,30].

In order to increase the understanding of the diversity of fungal viruses and explore potential biological control resources, strains which were isolated from sclerotia from infected rapeseed plants in a small field located at Xinyang City, Henan Province, China were used for RNA-Seq analysis to mine novel mycoviruses. After sequencing and assembling, a putative protein encoded by contig 2 (with a length of 11,864 nt) showed high similarity to endornaviral RNA polymerase, inferring that an unknown endornavirus may exist in a debilitating strain XY79. In this study, we determined the genome characteristics of this novel endornavirus and found great phylogenetic diversity of members in the family *Endornaviridae*.

## 2. Materials and Methods

### 2.1. Fungal Isolates and Biological Characterization

Sclerotia of *S. sclerotiorum* collected from diseased rapeseed plants growing in a small field in Xinyang City, Henan Province, China, were surface disinfected with 75% alcohol for 45 s and then washed with sterilized water twice. After air drying, sclerotia were incubated on potato dextrose agar (PDA) at 20 °C to perform fungal isolation. A total of 225 *S. sclerotiorum* isolates were used for virome analysis. A debilitating strain XY79 infected with a novel endornavirus was selected from 225 *S. sclerotiorum* strains for further study based on virome data. Reference strain 1980 was used as control.

To determine the growth rate, both strains XY79 and 1980 were inoculated on 20 mL PDA medium and incubated at 20 °C. The colony diameter was recorded at 24 and 36 h postinoculation (hpi). Growth rate of each strain was calculated. Colony morphology on PDA was photographed at 3, 5, and 12 days postinoculation (dpi).

To detect the virulence of strain XY79, detached rapeseed (*Brassica napus* cv. Huashuang No. 4) leaves with similar growth stage were inoculated with activating hyphal agar plug (5 mm diameter), and incubated at 20 °C with 95% relative humidity. The diameter of induced lesions on leaves at 36, 48, and 60 hpi was measured. Strain 1980 was used as control. Each strain had four replications, and the experiment was repeated twice.

### 2.2. Total RNA Extraction and Sequencing

Activated 225 *S. sclerotiorum* strains isolated from Xinyang City were inoculated onto cellophane-covered PDA plate. After 2–4 days of incubation, 0.1 g of mycelia was harvested and ground to a fine powder in liquid nitrogen for each strain. Total RNA extraction was carried out following the manual of the RNA extraction kit (Newbio industry, Tianjin, China). The concentration and quality of RNA were detected by Thermo Scientific^TM^ NanoDrop 2000 Spectrophotometer (Wilmington, DE, USA) and agarose gel electrophoresis. Then, RNA samples were mixed and stored at −80 °C before use. RNA-Seq for a mix RNA sample of strains from Xinyang city was performed by GENEWIZ Technology Services (Suzhou, China). After sequencing, viral contigs were assembled as previously described [31]. Clean reads were obtained by filtering out adapter-polluted, contaminated, paired-end reads shorter than 100 bp, low quality, high content of unknown base (N) reads, and the RNA and DNA sequences of *S. sclerotiorum* from raw data. Then, sequence assembly was conducted using CLC Genomics Workbench (version: 6.0.4). Final UniGenes generated from Primary UniGenes after splicing with CAP3 EST were subjected to BLAST, using BLASTx to search for homology with viral sequences against nonredundant (NR). To generate viral fragments, contigs corresponding to the same viruses were assembled by DNAMAN.

### 2.3. RT-PCR and RACE Cloning of Viral Genome

In order to detect the contigs obtained by RNA-Seq, the total RNA of strain XY79 was used to synthesize cDNA library by reverse transcription kit (Transgen, Beijing, China) with random primers. Viral specific primers were designed to detect viral contigs in the cDNA library (Table S1). PCR products were checked by agarose gel electrophoresis. 

To determine the terminal genome sequences of the novel endornavirus, rapid amplification of cDNA end (RACE) was conducted as previously described [32]. Briefly, the ends of total RNA were ligated with the linker RACE-OLOGO: 5’-p GCATTGCATCATGATCGATCGAATTCTTTAGTGAGGGTTAATTGCC-(NH2)-3’ by T4-RNA ligase (Axygen, Wujiang, China). The ligated RNA sample was then reverse transcribed into cDNA with primer RACE1 (5’-p-GGCAATTAACCCTCACTAAAG-3’). Using the cDNA as template, PCR was performed with primer pairs of RACE2 (5’- p-TCACTAAAGAATTCGAT -3’) and F1 or R1, corresponding to the 5’- or 3’-terminal sequences of the virus, respectively. Then, nest PCR was carried out to amplify the 5’ or 3’ ends using primer pairs RACE3 (5’-p-CGATCGATCATGATGCAATGC-3’) and F2 or R2, respectively. PCR products were purified with a gel extraction kit (Omega Bio, GA, USA) and then cloned into pMD19-T vector (TaKaRa, Dalian, China). The insertion fragments were sequenced by Biotechnology (Tyhygene, Wuhan, China). The experiment was repeated twice. The primers used are listed in Table S1.

### 2.4. Sequence Analysis

BLASTx was performed to find homologous amino acid (aa) sequences of viral contigs on NCBI website (http://blast.ncbi.nlm.nih.gov/Blast.cgi, accessed on 20 May 2021) (Table S2). ORFs were predicted with DNAMAN [33]. MOTIF Search (http://www.genome.jp/tools/motif, accessed on 20 May 2021) was used to predict putative viral protein. To obtain more classified endorna-like viruses that have similarity to the novel endornavirus found in this study, 500 of Max target sequence for general parameters in Algorithm parameters were selected to perform BLASTx. After BLASTx, endornaviruses and endorna-like viruses which have complete RdRp domain were selected. Alignment of amino acid sequences of viral RdRp domain was conducted using Mattf with Defaults program in software Jalview (Table S3). Identity matrix diagram of aa sequence of RdRp domain was carried out online using Clustal Omega program (https://www.ebi.ac.uk/Tools/msa/clustalo/, accessed on 20 May 2021) and RStudio. Phylogenetic tree based on the aa sequence of RdRp domain of selected viruses was constructed by the maximum likelihood (ML) method using IQ-Tree with 1000 bootstrap replications [34] (Table S3). Software IBS 1.0 was used to construct viral schematic diagram.

## 3. Results

### 3.1. Virus Diversity and Biological Characteristics of Strain XY79

**Figure 1:**
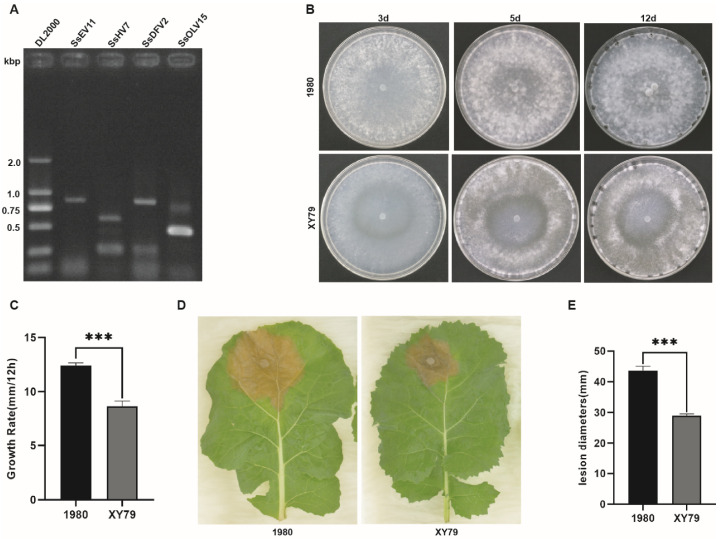
The biological characteristics of strain XY79 and virus detection using RT-PCR. (**A**) Detection diversity of viruses in the strain XY79. Lane DL2000, DNA marker; Lane 1 (845bp), SsEV11 (Sclerotinia sclerotiorum endornavirus 11); Lane 2 (566bp), SsHV7 (Sclerotinia sclerotiorum hypovirus 7); Lane 3 (846 bp), SsDFV2 (Sclerotinia sclerotiorum deltaflexivirus 2-WX); Lane 4 (439bp), SsOLV15 (Sclerotinia sclerotiorum ourmia-like virus 15). (**B**) The colony morphology of strains XY79 and 1980 (as control strain) on PDA plate at 20 °C. (**C**) The growth rate of strains XY79 and 1980 on PDA plate at 20 °C. The growth rate was measured by recording the colony diameter at 24 h and 36 h (*** *p* < 0.001). (**D**) Pathogenicity test, lesion induced by strains XY79 and 1980 on detached rapeseed leaf. The pictures were taken at 60 h postinoculation (hpi). (**E**) The difference in pathogenicity was calculated by recording the diameter of lesions on rapeseed leaves at 60 hpi (*p* < 0.001).

Via virome sequencing, a total of 65,989,085 raw reads were obtained. After removal of unqualified reads, sequence assembly and BLAST analysis, 41 contigs representing 41 different mycoviruses were generated from 225 *S. sclerotiorum* strains. Among them, Contig2 with a length of 11,864 nt, a fragment of a novel endornavirus, which showed the highest nucleotide sequence identity (45%) with Sclerotinia minor endornavirus 1 (SmEV1), was designated as Sclerotinia sclerotiorum endornavirus 11 (SsEV11). Using RT-PCR with contig2 specific primers, SsEV11 was found in a debilitating strain XY79. Thus, this strain was selected for further study. The mapping rates of reads of contig2 in sequencing data was 100%. The depth of sequencing was over 36x. Using RT-PCR with 41 viral specific primer pairs, 4 contigs, which corresponded to 4 different mycoviruses, were detectable in strain XY79. Thus, strain XY79 harbored four viruses, including a novel endornavirus and three reported viruses, Sclerotinia sclerotiorum hypovirus 7, Sclerotinia sclerotiorum deltaflexivirus 2-WX, and Sclerotinia sclerotiorum ourmia-like virus 15 (Figure 1A). Sclerotial formation and maturation of strain XY79 was delayed on PDA (Figure 1B). Growth rate of strain XY79 was 8.7 mm/12 h, which was significantly lower than that of strain 1980 (12.5 mm/12 h) (Figure 1C). Strain XY79 could induce lesions with an average diameter of 28.9 mm on detached rapeseed leaf. However, the lesion size was significantly smaller than those induced by strain 1980, whose average diameter was 43.7 mm (Figure 1D). The results indicated that strain XY79 was hypovirulent (Figure 1E).

### 3.2. Genome Characteristics of SsEV11

**Figure 2:**
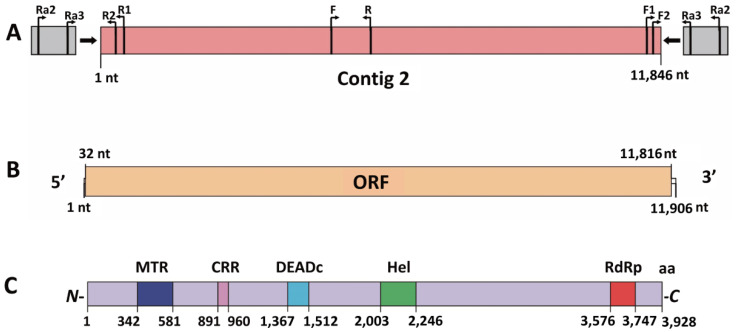
Diagrammatic sketch of the genome organization of SsEV11. (**A**) The information of Contig 2 obtained by virome sequencing and specific primers used for viral detection and rapid amplification of cDNA ends (RACE)-. The gray areas on both sides represent the RACE-OLOGO ligated using RACE method.Arrows represent direction and positions of the primers; F, F1 and F2 are primers ERV-F, ERV-F1 and ERV-F2; R, R1 and R2 are primers ERV-R, ERV-R1 and ERV-R2; Ra2 and Ra3 are primers Race2 and Race3. (**B**) The genome organization of SsEV11, showing 5′ and 3′ untranslated regions (UTR) and open reading flame (ORF) region. (**C**) The polyprotein encoded by the ORF of SsEV11 and its conserved function domains MTR, CRR, DEADc, Hel and RdRp. the Arabic numerals show the amino acid position of each conserved domain.

Based on the sequence of contig 2, the 5’- and 3’- terminal sequences of SsEV11 were amplified by RACE PCR (Figure 2A). The 5’ untranslated region (UTR) is 31 nt in length, while the 3’ UTR is 90 nt (Figure 2B). Viral genome was assembled with DNAMAN program. The complete genome sequence of SsEV11 is 11,906 nt of 39.2% GC content with one large ORF putatively encoding a polyprotein of 3928 amino acid residues. The genomic sequence of SsEV11 was submitted to GenBank database and assigned accession number MZ605432.

**Figure 3:**
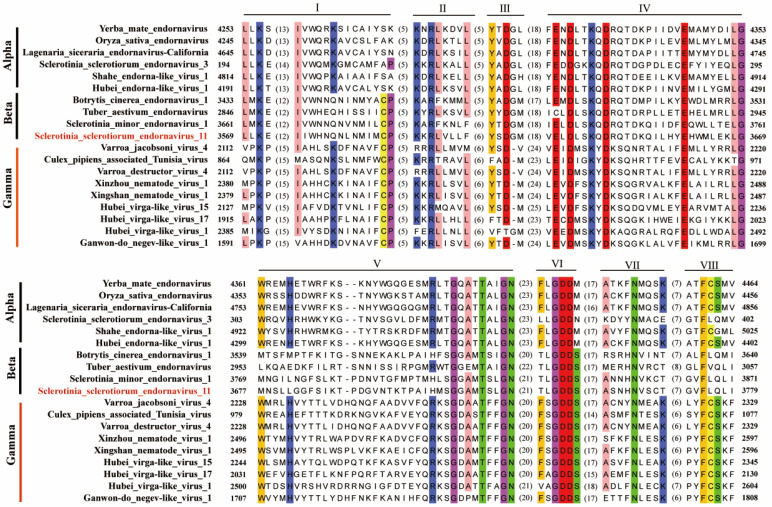
Alignment of RdRp domain of selected endornaviruses and endorna-like viruses. Conserved motifs are marked by Roman numerals from I to VIII. “Alpha” and “beta” represent alphaendornavirus and betaendornavirus, respectively; “Gamma” represents proposed gammaendornaviruses or endorna-like viruses. Novel endornavirus is highlighted with red characters.

Using MotifFinder website, the polyprotein was predicted to contain a viral MTR domain from 342 aa to 581 aa, a cysteinerich region (CRR) from 891 aa to 960 aa, a putative DEADc from 1376 aa to 1514 aa, a viral Hel domain from 2003 aa to 2246 aa, and an RdRp_2 domain from 3576 aa to 3747 aa (Figure 2C). The RdRp_2 domain of SsEV11 shared eight typical conserved motifs (I–VIII) with other endornaviruses or endorna-like viruses, which included viruses from the genera *Alphaendornavirus* and *Betaendornavirus*, and endorna-like viruses (Figure 3) [12,35,36].

### 3.3. Alignment Analysis of SsEV11

According to the results of BLASTp search based on polyproteins sequence, SsEV11 showed the highest identity (45.61%) with Sclerotinia minor endornavirus 1 (SmEV1), the exemplar strain of *Sclerotinia minor betaendornavirus 1* [37]. The polyproteins of SsEV11 also shared 35% identity with that of Gremmeniella abietina type B RNA virus XL1 (GaBRV/XL1) [38], the exemplar isolates of *Gremmeniella abietina betaendornavirus 1*, and 32% aa identity with that of Tuber aestivum endornavirus (TaEV) [39], the exemplar isolate of *Tuber aestivum betaendornavirus* (Table 1). The RdRp domain of SsEV11 shares 68.94%, 48.51%, and 43.40% aa identity with those of SmEV1, GaBRV/XL1, and TaEV, respectively (Table 1). The result of BLASTn search showed that the whole nucleotide sequence of SsEV11 share no significant identity to any reported viruses. However, some fragments of SsEV11 shares 68% identity (988/1461) with Botrytis endornavirus 1 [40], 67% identity (747/1122) with that of Sclerotinia sclerotiorum endornavirus 3 (SsEV3), and 65% identity (898/1373) with SmEV1 (Table 1). Thus, SsEV11 represents a novel species in the genus *Betaendornavirus*.

### 3.4. Phylogenetic Analysis Exhibits Multiple Lineages of Endornaviruses

**Figure 4:**
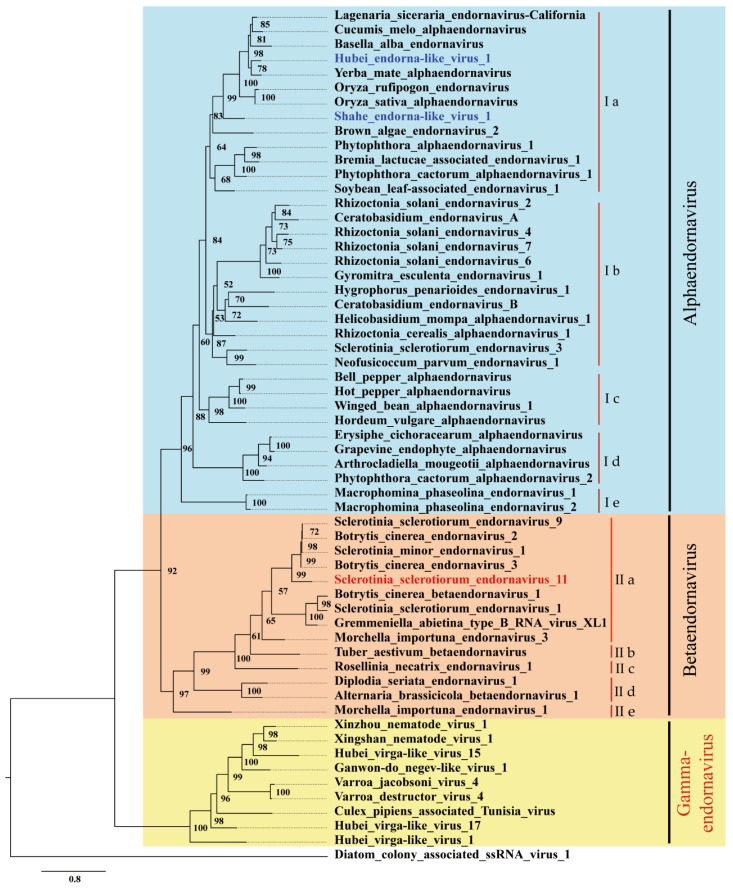
Phylogenetic analysis of SsEV11 and other selected endornaviruses and endorna-like viruses based on the RdRP domain using Maximum Likelihood program with 1000 bootstrap replicates. GenBank accession numbers and virus names are listed in Table S3. Viruses written in blue are those infecting or associated with insects, written in red is newly identified virus.

**Figure 5:**
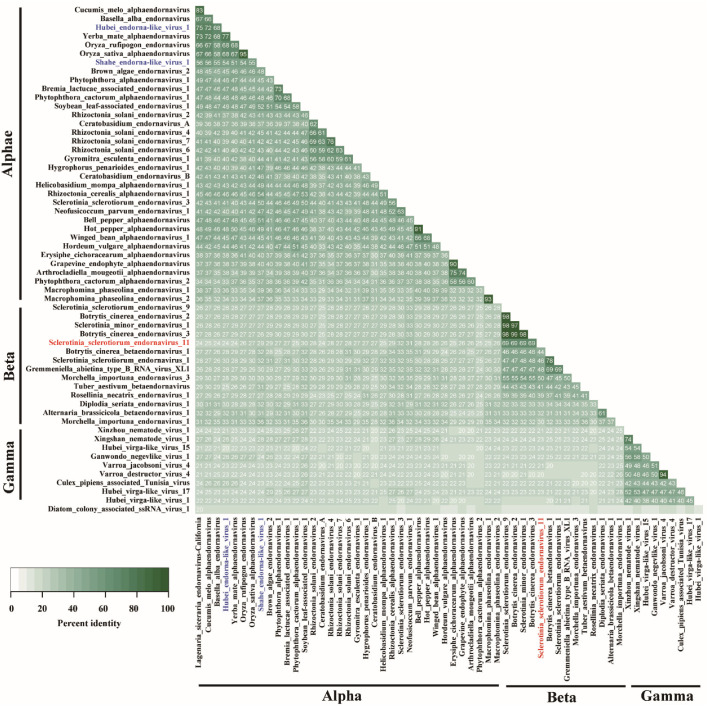
Matrix diagram of amino acid identities of RdRp domain among selected endornaviruses and endornalike viruses by using Clustal Omega 2.1. Alpha, Beta, and Gamma represent alphaendornaviruses, betaendornaviruses, and proposed gammaendornavirus, respectively; the cutoff values were 25%. The information of selected viruses and their RdRp domains are listed in Table S3. Viruses written in blue are viruses that infect insects. Newly identified endornavirus is written in red. Alignment analysis was carried out on website https://www.ebi.ac.uk/Tools/msa/clustalo/, accessed on 20 May 2021.

Polyprotein sequence of SsEV11 was used as seed to blast NCBI database. Then, aa sequences of endornaviruses and endorna-like viruses that had completed RdRp domain were downloaded. RdRp domain of these viruses (Table S3) was used to perform phylogenetic analysis with IQ-Tree. Three large clusters, Cluster I, Cluster II, and Cluster III, were observed in phylogenetic tree (Figure 4). The result of multiple sequence alignment analysis also confirmed that three large lineages present in endornavirus (Figure 5).

Cluster I includes typical species of *Alphaendornavirus*, unclassified viruses that isolated from fungi, oomycetes, and insects, such as Hubei endorna-like virus 1 and Shahe endorna-like virus 1 [14]. It showed highly phylogenetical diversity. Combining the results of multiple sequence alignment analysis (Figure 5), Cluster I could be divided into five subclusters, designated as Cluster Ia, Ib, Ic, Id, and Ie. Cluster Ia had 13 members, which were isolated from plants (mostly), oomycetes, brown algae, and insects; Cluster Ib had 12 members, all of which were isolated from fungi of basidiomycetes and ascomycetes; Cluster Ic had 4 members, all of them were associated with plants; Cluster Id had 4 members isolated from fungi, oomycetes, or associated to plants; and Cluster Ie had 2 members, which were isolated from fungi of ascomycetes.

Cluster II includes typical species of *Betaendornavirus*, SsEV11 and other unclassified endornaviruses, all of which were isolated from ascomycetes. However, Cluster II could also be further divided into five subclusters, designated as Cluster IIa, IIb, IIc, IId, and IIe. Cluster IIa had nine members, including members of *Betaendornavirus* and SsEV11 which was closely related to SsEV9, SmEV1, BcEV2, and BcEV3, but formed a separate branch; Cluster IIb and Cluster IIc had one member, respectively, namely Tuber aestivum endornavirus and Rosellinia necatrix endornavirus 1 [39,41]; Cluster IId had two members, Diplodia seriata endoranvirus 1 and Alternaria brassicicola betaendornavirus 1 [42,43]; and Cluster IIe had only one member, Morchella impotuna endornavirus 1 [44].

Cluster III included endorna-like viruses, which were isolated from insects, nematodes, and mites. Viruses in Cluster III were relatively distant to these of Cluster I and Cluster II. Multiple alignment analysis showed that these viruses were closely related to each other (Figure 5). In addition, RdRp domains of SsEV11 and these viruses were similar (Table 2). Thus, we proposed to construct a novel genus, Gammaendornavirus, in the family *Endornaviridae* to accommodate the viruses in the Cluster III.

## 4. Discussion

In this study, we characterized a novel endornavirus, Sclerotinia sclerotiorum endornavirus 11 (SsEV11), that infected *S. sclerotiorum* strain XY79. Complete nucleotide sequence of SsEV11 had no significant identity to other viruses, even at the most similar parts (such as sequence coding for RdRp). Nucleotide identity between SsEV11 and other viruses were less than 75% (Table 1), suggesting that SsEV11 represents a novel species in the genus *Betaendornavirus*. This species, SsEV11, was tentatively designated as Sclerotinia sclerotiorum betaendornavirus 2. Phylogenetically, Sclerotinia sclerotiorum betaendornavirus 2 is closely related to *Sclerotinia minor betaendornavirus 1*.

*S. sclerotiorum* hosts both alphaendornaviruses and betaendornaviruses. Besides SsEV11, so far, 16 endornaviruses, isolated from *S. sclerotiorum*, were recorded in NCBI database (Table S4). Four sequences belong to Sclerotinia sclerotiorum endornavirus 1 (SsEV 1), three belong to Sclerotinia sclerotiorum endornavirus 2 (SsEV 2). SsEV 1 and SsEV 2 are the same species, *Sclerotinia sclerotiorum betaendornavirus 1*, due to the nt identity of genome RNA between SsEV1 and SsEV2 is more than 81%. Two sequences belong to Sclerotinia sclerotiorum endornavirus 3 (QOE77938.1 and AWY10956.1), but they are fully different. Sclerotinia sclerotiorum endornavirus 3/SX276 (QOE77938.1), which was isolated from *S. sclerotiorum* strain in China, is a betaendornavirus, while Sclerotinia sclerotiorum endornavirus 3 (AWY10956.1), which was associated with *S. sclerotiorum* strain in Australia, is a member of *Alphaendornavirus.* RdRp aa sequences of Sclerotinia sclerotiorum endornavirus 3 (AWY10956.1) shares 59% (310/529) aa identity and 73% similarity (389/529) to that of Neofusicoccum parvum endornavirus 1 [45], which is located on the Cluster Ib (Figure 4). Sclerotinia sclerotiorum endornavirus 4, −5, −6, −7, 8, −9, −10 have been reported with incomplete genome sequence. The known sequence of Sclerotinia sclerotiorum endornavirus 4 is partial, which is absent of the sequence coding for RdRp domain, the incomplete aa sequence shows 31% (1104/3520) aa identity and 49 % (1758/3520) similarity to that of Neofusicoccum parvum endornavirus 1. Thus, SsEV4 represents a novel species in the genus *Alphaendornavirus*. Sclerotinia sclerotiorum endornavirus 5 may represent a new species in the genus *Alphaendornavirus* because its known sequence shows 74.27% (153/206) aa identity to that of SsEV 4. Taken together, three endornaviruses, infecting *S. sclerotiorum*, are closely related to Neofusicoccum parvum endornavirus 1. SsEV 6 shares 48% aa identity to Helicobasidium mompa alphaendornavirus 1, which could be grouped in *Alphaendornavirus*, Cluster Ic (Figure 4) [35]. Sclerotinia sclerotiorum endornavirus 7 (SsEV 7) and Sclerotinia sclerotiorum endornavirus 9 (SsEV 9) are the same virus, which belong to the species *Sclerotinia minor betaendornavirus 1*. Sclerotinia sclerotiorum endornavirus 8 (SsEV 8) is an isolate of *Botrytis cinerea betaendornavirus 1*, since the nt identity between them is more than 82%. Sclerotinia sclerotiorum endornavirus 10 shares 55% aa identity to Sclerotinia sclerotiorum endornavirus 2. Thus, *S. sclerotiorum* hosts various endornaviruses both on genus and species levels (Table S4).

The members of genus *Alphaendornavirus* were originally identified from plants, and later, were also found to infect fungi and oomycetes. Here, we found an endornavirus that infects brown algae (Brown algae endornavirus 2) and two viruses associated with insects (Shahe endorna-like virus 1 and Hubei endorna-like virus 1) [14,46] are members of *Alphaendornavirus*. Brown algae endornavirus 2 are closely related to Shahe endornalike virus 1 [14]. Hubei endorna-like virus 1 and Yerba mate alphaendornavirus have a close evolutionary relationship. Shahe endorna-like virus 1 is closely related to Oryza sativa endornavirus and Oryza rufipogon endornavirus (Figure 4) [4,47]. Thus, these endornaviruses should be members of *Alphaendornavirus*. In addition, this conclusion was also supported by alignment analysis (Figure 3 and Figure 5). Therefore, the host range of viruses in the genus *Alphaendornavirus* can be expanded to algae and insects.

Previously, a novel genus in the family *Endornaviridae* was proposed based on a novel Rhizoctonia solani endornavirus [48]. As aa identity of RdRp domain of this virus share quite high identity to alphaendornavirus, we suggested that this virus is actually an alphaendornavirus (Figure 4 and Figure 5). However, the members in *Alphaendornavirus* are phylogenetically diverse. At least, five lineages (Cluster Ia–Ie) could be grouped. Endornaviruses that infect plants distribute in three subclusters (Cluster Ia, Ic, and Id), suggesting that these plant infecting endornaviruses have different origins. The aa identity of RdRp domains of viruses in Cluster Id and Cluster Ie are considerably low compared to those of viruses in other subclusters. For example, the aa identities are less than 40% for Cluster Ie, and less than 42% for Cluster Id (Figure 5). Therefore, it is likely that both Cluster Id and Cluster Ie may represent novel genera which are phylogenetically closely related to *Alphaendornavirus*. 

A similar diverse situation has also been observed in the genus *Betaendornavirus*. In Cluster II, viruses which belong to Cluster IIc, Cluster IId, and Cluster IIe may represent three novel genera, which are related to betaendornaviruses, respectively (Figure 4). It is worth noting that all viruses in Cluster II were isolated from ascomycetes. However, it does not mean that ascomycetes could only be infected by betaendornavirus. For example, *S. sclerotiorum* actually could be infected by either alphaendornaviruses or betaendornaviruses (Table S4).

Viruses in Cluster III are phylogenetically closely related to typical alphaendornaviruses and betaendornaviruses (Figure 4 and Table 2). These viruses are mainly isolated from various animals, such as nematodes, mites, and insects (Table 2). They are tightly related to endornaviruses and have the feature domains of RdRp of endornaviruses (Figure 3 and Figure 5; Table 2). For example, Xingshan nematode virus 1 shows 30% (73/243) aa identity and 44% (109/243) aa similarity of RdRp domain with Hot pepper alphaendornavirus in *Alphaendornaviru*, and shows 30% (52/172) aa identity and 46% (80/172) aa similarity with Botrytis cinerea betaendornavirus 1 in *Betaendornavirus*, respectively [49,50]. Another species, Hubei virga-like virus 17, shows 30% (63/211) aa identity and 43% (92/211) aa similarity with Brown algae endornavirus 2, a member of *Alphaendornavirus*. Furthermore, the virus also shows 30% (70/233) aa identity and 46% (108/233) aa similarity with Morchella importuna endornavirus 1, a member of *Betaendornavirus* [44,46]. Therefore, we proposed a novel genus Gammaendornaviruses in the family *Endornaviridae*. The establishment of Gammaendornavirus may deepen our understanding on the diversity and evolution of endornaviruses, and may facilitate to investigate the connection among endornaviruses which originally isolated from fungi, plants, oomycetes, and animals.

Endornaviruses are usually not associated with hypovirulence and do not affect the phenotype of their host [6,51,52]. However, so far, more and more endornaviruses with biocontrol potential have been reported. Horizontal transmission of the Rhizoctonia solani endornavirus 1 (RsEV1) can lead to attenuation of the derivative isogenic strain of the virulent strain GD-118P [36]. Further, Yang et al. found that Sclerotinia minor endornavirus 1 (SmEV1) has horizontal and vertical transmission characteristics, and the mycelium fragments of strain LC22 can attenuate the virulence of *S. minor* [37]. Recent studies have found that plants infected by Bell pepper endornavirus (BPEV) have alterations of organelles and other cell components, which are believed to be caused by parasitic effects between the endornavirus and host [53]. Additionally, the coinfection of Phytophthora endornavirus 2 (PEV2) and Phytophthora endornavirus 3 (PEV3) also affects the sensitivity of oomycetes to fungicides [54]. Here, we found that the SsEV11-infecting strain XY79 has some abnormal phenotypes, such as lower growth rate on PDA medium, delayed sclerotial formation and maturation, and decreased virulence on detached rapeseed leaves. However, weather these attenuated traits were contributed by SsEV11 is unknown since XY79 is co-infected by other viruses.

## Figures and Tables

**Table 1:** BLASTp analysis of polyprotein, RdRp, Hel, and Mtr domains between Sclerotinia sclerotiorum endornavirus 11 and selected endornaviruses.

Virus Name	Abbreviation	GenBank Accession No	Genome Length (nt)	Host	Sequence Identity (%)	
					Polyprotein	RdRp
Botrytis cinerea endornavirus 3	BcEV3	MN839443	13,582	F	44.66	68.94
Botrytis cinerea endornavirus 2	BcEV2	MN617758	13,581	F	44.75	68.51
Sclerotinia sclerotiorum endornavirus 9	SsEV9	MT646421	13,562	F	44.76	69.36
Sclerotinia minor endornavirus 1	SmEV1	NC_040631	12,626	F	45.61	68.94
Gremmeniella abietina type B RNA virus XL1	GaBRV/XL1	NC_007920	10,375	F	35.00	48.51
Sclerotinia sclerotiorum endornavirus 1	SsEV1/JZJL2	NC_021706	10,770	F	29.00	45.53
Botrytis cinerea endornavirus 1	BcEV1	NC_031752	11,557	F	29.08	43.40
Tuber aestivum endornavirus	TaEV	NC_014904	9760	F	32.00	43.40
Rosellinia necatrix endornavirus 1	RnEV1	NC_030938	9639	F	30.11	42.11
Alternaria brassicicola endornavirus 1	AbEV1	NC_026136	10,290	F	27.59	32.91
Grapevine endophyte endornavirus	GEEV	NC_019493	12,154	P	24.00	26.89
Bell pepper endornavirus	BpEV	NC_015781	14,728	P	24.00	29.86
Winged bean alphaendornavirus 1	WbEV1	NC_031336	14,623	P	23.39	25.45
Rhizoctonia solani endornavirus—RS002	RsEV/RS002	KC792590	14,694	F	29.00	29.20
Hot pepper endornavirus	HpEV	NC_027920	14,729	P	25.20	28.77
Oryza sativa endornavirus	OsEV	D32136	13,952	P	25.00	28.70
Oryza rufipogon endornavirus	OrEV	NC_007649	17,635	P	24.00	29.24
Phaseolus vulgaris endornavirus 1	PvEV1	AB719397	13,908	P	24.00	23.85
Phytophthora endornavirus 1	PEV1	AJ877914	13,883	O	26.68	27.44

F, fungus; P, plant; O, oomycete.

**Table 2:** BLAST analysis of the RdRp domain of SsEV11 and selected endorna-like viruses.

Virus Name	Host	Accession Number	Genome Length (nt)	Query Cover	Identity (%)	Positivity (%)
Varroa jacobsoni virus 4	mite	QKW94174	8337	84%	24% (51/216)	47% (102/216)
Culex pipiens associated Tunisia virus	insects	AUT77208	6816	88%	26% (55/215)	44% (95/215)
Varroa destructor virus 4	mite	QGA69815	8332	84%	22% (45/204)	47% (96/204)
Xinzhou nematode virus 1	nematode	NC_033728	11,525	83%	28% (57/207)	47% (99/207)
Xingshan nematode virus 1	nematode	NC_032483	11,374	76%	29% (57/198)	47% (94/198)
Hubei virga-like virus 15	insects	NC_033211	10,423	76%	28% (53/188)	49% (93/188)
Hubei virga-like virus 17	insects	NC_033222	9481	72%	29% (53/180)	45% (82/180)
Hubei virga-like virus 1	insects	NC_033165	9141	66%	26% (43/163)	49% (81/163)
Ganwon-do negev-like virus 1	mite	MT757507	6451	88%	26% (58/221)	43% (97/221)
Hubei endorna-like virus 1	insects	NC_033204	13,693	88%	27% (57/210)	48% (102/210)
Shahe endorna-like virus 1	insects	NC_032798	15,783	79%	33% (64/192)	53% (103/192)

BLAST analysis was carried out on https://www.ncbi.nlm.nih.g, accessed on 20 May 2021.

## Data Availability

Not applicable.

## Supplementary Materials

The following are available online at https://www.mdpi.com/article/10.3390/v14030456/s1. Table S1: The information of primers used in this study. Table S2: Information of four viral contigs in strain XY79. Table S3: The information of RdRp domain of viruses selected for multiple sequence alignment analysis and phylogenetic analysis in this study. Table S4: Summary of endornaviruses that infect *Sclerotinia sclerotiorum*.
