# Supplementary material for: Genome Characterization and Phylogenetic Analysis of a Novel Endornavirus That Infects Fungal Pathogen Sclerotinia sclerotiorum
Source: Viruses. 2022 Feb 23;14(3):456. doi: 10.3390/v14030456 (PMC8953294; doi:10.3390/v14030456)
Supplement: Supplementary file 1 [file viruses-14-00456-s001.zip › viruses-1578821-supplementary.pdf]

viruses

MDPI

Supplementary Materials

Table S1. The information of primers used in this study.

Primer name	Primer Sequence
ERV-F	ACATCTCATTTCCACTACCCGC
ERV-R	ATAGGTAGCGATGGTTGCGAAG
ERV-F1	TCACGCAAATCACTAAGGCTG
ERV-F2	TTCTGTCTCAATGCTTGGGTCAC
ERV-R1	GAACAACATTTGAAGAAAGACTCC
ERV-R2	ACAATACAAGGTGTTGGAGTTATACGG
Contig12-F	TATGGCGTCTACAGCAGCGG
Contig12-R	ACTTTACTGGCTCCTTTGACCCTG
Contig87-F	AAACAGTGGCGTAACGGTCG
Contig87-R	ATGCTGATGTCGTGGTCCTCC
Contig169-F	GTTGTTTCGGCGAATGCTTG
Contig169-R	TAAGTGCTGCCTCACGAACG

Table S2. Information of four viral contigs in strain XY79.

Contig Number	Length (bp)	Best Match (Blastx)	Accession	Identity	Query Cover	Order or Family
Contig 2	11846	Sclerotinia minor endornavirus 1	NC_040631.1	45.61%	83%	Endornaviridae
Contig 12	8428	Sclerotinia sclerotiorum hypovirus 7	MT646394.1	99.34%	96%	Hypoviridae
Contig 87	5756	Sclerotinia sclerotiorum deltaflexivirus 2-WX	MT646426.1	99.63%	98%	Deltaflexiviridae
Contig 169	3261	Sclerotinia sclerotiorum ourmia-like virus 15	MT646399.1	97.69%	67%	Botourmiaviridae

Table S3. The information of RdRp domain of viruses selected for multiple sequence alignment analysis and phylogenetic analysis in this study.

Virus Name	Accession	Polyprotein (aa)	RdRp(aa)	
			Begin	Length
Oryza rufipogon endornavirus	YP_438202.1	4627	4136	237
Oryza sativa endornavirus	BAA06862.1	4572	4081	237
Hubei endorna-like virus 1	YP_009337681.1	4518	4081	237
Yerba mate endornavirus	YP_009046830.1	4580	4089	237
Lagenaria siceraria endornavirus -California	YP_009010973.1	4974	4481	236
Cucumis melo endornavirus	ALV83885.1	4939	4449	236
Basella alba endornavirus 1	AB844264.1	4658	4162	237
Shahe endorna-like virus 1	YP_009336626.1	5144	4681	237
Brown algae endornavirus 2	BBZ90074.1	4168	3721	237
Soybean leaf-associated endornavirus 1	ALM62234.1	1293	841	237
Phytophthora endornavirus 1	CAI47561.1	4612	4121	237
Bremia lactucae associated endornavirus 1	QIP68005.1	4405	3901	237
Phytophthora cactorum alphaendornavirus 1	QUE45702.1	404	61	237
Rhizoctonia solani endornavirus 2	YP_010086587.1	5262	4861	248
Ceratobasidium endornavirus A	YP_009310113.1	4968	4561	252
Rhizoctonia solani endornavirus 6	QDW65433.1	5077	4681	248

Gyromitra esculenta endornavirus 1	AZT88615.1	4846	4261	252
Rhizoctonia solani endornavirus 4	QDW65431.1	6719	6301	252
Rhizoctonia solani endornavirus 7	QDW65434.1	4757	4381	255
Rhizoctonia cerealis endornavirus 1	AGY34962.1	5747	5314	237
Rhizoctonia solani endornavirus-RS002	AHL25280.1	4893	4561	177
Sclerotinia sclerotiorum endornavirus 3	AWY10956.1	529	61	238
Neofusicoccum parvum endornavirus 1	QTE76046.1	4594	4081	238
Helicobasidium mompa endornavirus 1	BAE94538.1	5373	4880	238
Ceratobasidium endornavirus B	YP_009310114.1	5744	5281	240
Hygrophorus penarioides endornavirus 1	QUP79367.1	5522	5101	239
Bell pepper endornavirus	YP_004765011.1	4815	4323	237
Hot pepper endornavirus	YP_009165596.1	4884	4392	237
Winged bean alphaendornavirus 1	YP_009305414.1	4785	4293	237
Hordeum vulgare endornavirus	ALT66307.1	4663	4170	237
Erysiphe cichoracearum alphaendornavirus	YP_009225663.1	3952	3421	236
Grapevine endophyte alphaendornavirus	YP_007003829.1	4027	3481	236
Arthrocladiella mougeotii alphaendornavirus	AZO92732.1	3871	3361	236
Phytophthora cactorum alphaendornavirus 2	QUA12641.1	4164	3721	237
Macrophomina phaseolina endornavirus 1	QOE55584.1	2721	2221	237
Macrophomina phaseolina endornavirus 2	QOE55585.1	2706	2221	237
Sclerotinia sclerotiorum endornavirus 9	QUE49189.1	4500	4021	235
Sclerotinia minor endornavirus 1	YP_009552723.1	4020	3541	235
Botrytis cinerea endornavirus 2	QJT73713.1	4501	3961	235
Botrytis cinerea endornavirus 3	QLC37097.1	4501	4021	235
Sclerotinia sclerotiorum endornavirus 11	MZ605432	3927	3409	235
Botrytis cinerea endornavirus 1	YP_009315910.1	3787	3270	233
Sclerotinia sclerotiorum endornavirus 1/JZJL2	YP_008169851.1	3491	2972	233
Gremmeniella abietina type B RNA virus XL1	YP_529670.1	3429	2915	232
Discula destructiva virus 3	AAK55403.1	238	1	103
Morchella importuna endornavirus 3	AZT88618.1	4734	4201	236
Tuber aestivum endornavirus	YP_004123950.1	3217	2681	237
Rosellinia necatrix endornavirus 1	YP_009276355.1	3148	2664	236
Diplodia seriata endornavirus 1	QDB74983.1	3318	2821	234
Alternaria brassicicola betaendornavirus 1	YP_009115493.1	3400	2881	234
Morchella importuna endornavirus 1	AZT88616.1	5447	4922	235
Xinzhou nematode virus 1	YP_009345041.1	2708	2161	241
Xingshan_nematode_virus_1	YP_009333310.1	2707	2161	241
Hubei_virga-like_virus_15	YP_009337693.1	2454	1921	242
Ganwon-do_negev-like_virus_1	QPN36965.1	1919	1441	241
Varroa_jacobsoni_virus_4	QKW94174.1	2442	1921	241
Varroa_destructor_virus_4	QGA69815.1	2442	1921	241
Hubei_virga-like_virus_17	YP_009337715.1	2239	1741	237
Culex_pipiens_associated_Tunisia_virus	AUT77208.1	1079	556	236
Hubei_virga-like_virus_1	YP_009337423.1	2712	2161	248

Table S4. Summary of endornaviruses that infect *S. sclerotiorum**.

Genus Name	Species Name (or Tentative Name)	Virus name	Acquired aa Sequence Length	Accession No.	Most Related Known Endornavirus /Accession No.	Identity
Alphaendornavirus	*Sclerotinia sclerotiorum alphaendornavirus 1*	Sclerotinia sclerotiorum endornavirus 6(SsEV6)	100	AWY10959	Helicobasidium mompa alphaendornavirus 1/ BAE94538.1	38% (40/105)
	Sclerotinia sclerotiorum alphaendornavirus 2	Sclerotinia sclerotiorum endornavirus 4(SsEV4)	3678	AWY10957	Neofusicoccum parvum endornavirus 1/ QTE76046.1	31% (1104/3520)
	Sclerotinia sclerotiorum alphaendornavirus 3	Sclerotinia sclerotiorum endornavirus 3	529	AWY10956	Neofusicoccum parvum endornavirus 1/ QTE76046.1	59% (310/529)
	Sclerotinia sclerotiorum alphaendornavirus 4	Sclerotinia sclerotiorum endornavirus 5	206	AWY10958	Neofusicoccum parvum endornavirus 1/ QTE76046.1	44% (89/204)
Betaendornavirus	*Sclerotinia sclerotiorum betaendornavirus 1*	Sclerotinia sclerotiorum endornavirus 1(SsEV1)	3491	YP_008169851	Sclerotinia sclerotiorum betaendornavirus 1/ YP_009022070.1	89% (3103/3490)
		Sclerotinia sclerotiorum endornavirus 1 (SsEV 1)	3491	AJF94392	Sclerotinia sclerotiorum betaendornavirus 1/ YP_009022070.1	88% (3088/3491)
		Sclerotinia sclerotiorum endornavirus 1-WX(SsEV 1/WX)	3491	QUE49181	Sclerotinia sclerotiorum betaendornavirus 1/ YP_009022070.1	89% (3107/3490)
		Sclerotinia sclerotiorum endornavirus 1-A(SsEV1 A)	836	AWY10954	Sclerotinia sclerotiorum betaendornavirus 1/ YP_009022070.1	86% (721/836)
		Sclerotinia sclerotiorum endornavirus 2(SsEV 2)	3459	AND83000	Sclerotinia sclerotiorum betaendornavirus 1/ YP_009022070.1	90% (3100/3458)
		Sclerotinia sclerotiorum betaendornavirus 1(SsEV2)	3459	YP_009022070	Sclerotinia sclerotiorum betaendornavirus 1/ YP_009022070.1	100% (3459/3459)
		Sclerotinia sclerotiorum endornavirus 2-A(SsEV 2A)	2469	AWY10955	Sclerotinia sclerotiorum betaendornavirus 1/ YP_009022070.1	88% (2205/2495)

Sclerotinia minor betaendornavirus 1	Sclerotinia sclerotiorum endornavirus 3(SsEV 3)	4184	QOE77938	Sclerotinia minor endornavirus 1/ YP_009552723.1	88% (3531/4020)
	Sclerotinia sclerotiorum endornavirus 7(SsEV 7)	183	AWY10960	Sclerotinia minor endornavirus 1/ YP_009552723.1	96% (176/183)
	Sclerotinia sclerotiorum endornavirus 9 (SsEV9)	4500	QUE49189	Sclerotinia minor endornavirus 1/ YP_009552723.1	88% (3363/3832)
Botrytis cinerea betaendornavirus 1	Sclerotinia sclerotiorum endornavirus 8 (SsEV)	3799	QUE49098	Botrytis cinerea betaendornavirus 1/ YP_009315910.1	87% (3297/3787)
Sclerotinia sclerotiorum betaendornavirus 2	Sclerotinia sclerotiorum endornavirus 11(SsEV11)	3928	MZ605432	Sclerotinia minor endornavirus 1/ YP_009552723.1	46% (919/2015)
Sclerotinia sclerotiorum betaendornavirus 3	Sclerotinia sclerotiorum endornavirus 10 (SsEV10)	835	QUE49166	Sclerotinia sclerotiorum endornavirus 2	52% (439/839)

* The aa sequences are acquired from https://www.ncbi.nlm.nih.gov (accessed on 15 January 2021) and carried out Blastp analysis. The most related viruses are listed.
